# Database of host-pathogen and related species interactions, and their global distribution

**DOI:** 10.1038/sdata.2015.49

**Published:** 2015-09-15

**Authors:** Maya Wardeh, Claire Risley, Marie Kirsty McIntyre, Christian Setzkorn, Matthew Baylis

**Affiliations:** 1 Department of Epidemiology and Population Health, Institute of Infection and Global Health, University of Liverpool, Leahurst Campus, Chester High Road, Neston, Cheshire CH64 7TE, UK; 2 Institute of Biological and Environmental and Rural Sciences, Aberystwyth University, Penglais Campus, Aberystwyth SY23 3DA, UK; 3 Health Protection Research Unit for Emerging and Zoonotic Infections, University of Liverpool, Leahurst Campus, Chester High Road, Neston, Cheshire CH64 7TE, UK

**Keywords:** Ecological epidemiology, Infectious diseases, Epidemiology, Databases

## Abstract

Interactions between species, particularly where one is likely to be a pathogen of the other, as well as the geographical distribution of species, have been systematically extracted from various web-based, free-access sources, and assembled with the accompanying evidence into a single database. The database attempts to answer questions such as what are all the pathogens of a host, and what are all the hosts of a pathogen, what are all the countries where a pathogen was found, and what are all the pathogens found in a country. Two datasets were extracted from the database, focussing on species interactions and species distribution, based on evidence published between 1950–2012. The quality of their evidence was checked and verified against well-known, alternative, datasets of pathogens infecting humans, domestic animals and wild mammals. The presented datasets provide a valuable resource for researchers of infectious diseases of humans and animals, including zoonoses.

## Background & Summary

Communicable diseases continue to impose a tremendous burden of mortality and morbidity on humans. The most recent assessment of the global burden of disease^1^ finds that communicable diseases were directly responsible for nearly nine million deaths in 2013, some 16% of the total. Communicable diseases were estimated to have caused nearly 565 million disability-adjusted life years (DALYs) in 2010, about 23% of the total^2^. DALYs are a measure that combines years of life lost (from premature death) and years lived with disability, and therefore takes account of both mortality and morbidity.

The global burden of communicable diseases of animals, including livestock, has not been quantified, but is undoubtedly high. Such diseases are important for four major reasons: (i) animal welfare; (ii) livestock provide income, food and clothing and, particularly in low income countries, building materials and draught power; as such, livestock diseases may affect human mortality and morbidity indirectly, affecting food security and contributing to malnutrition; (iii) animal diseases are a source of human disease. About three-fifths of human diseases are believed to have arisen from animal pathogens, especially those of livestock^3–5^. Effort is underway to estimate the global burden of human disease that is food-borne (i.e., originates from animals and is transmitted in food^6^); (iv) communicable diseases may limit or cause extreme fluctuations in population size of wild animals^7^ and reduce the chances of survival of endangered or threatened species^8^. Indeed, disease can be the primary cause of extinction in animals or be a significant contributory factor towards it.

Despite the importance of communicable diseases, there have been few attempts to construct datasets of the full set of infectious agents of humans or animals. Such datasets have many uses, for example facilitating the estimation of comprehensive infectious disease burdens, quantifying important characteristics of diseases, such as type of agent, whether they are zoonotic etc.; and providing a baseline for studying the rate of emergence of new diseases. The most notable attempt that has been made^5^ found 1415 pathogens of humans. The data were compiled from fourteen textbooks of human and zoonotic disease and twenty-five reviews of emerging diseases. These sources were not systematically selected or exhaustive and may themselves have had biases and gaps in the diseases listed. The final dataset is also time-limited and does not reference communicable diseases that have emerged since publication. There is no comprehensive dataset of animal diseases.

Here we provide a full description of the development of a database of the interactions of species with (i) other species and (ii) locations. A large subset of the database is interactions where one species causes disease in another, i.e., pathogen-host relationships. However, a number of other interactions are also included such as commensal/mutualistic and vector-host. The database has been populated using automated procedures from freely-available, web-based sources. The database stores information on species (including hosts and pathogens) and where they have been found in the world (at national and sub-national [i.e., state or regional] level). Sources of supporting evidence are linked to each entry. Importantly, the database can be regularly updated and is free to access.

We finish by describing the validation of three subsets of the database (namely, species that are recorded interacting with humans, domestic animals, and wild mammals) with lists of pathogens of these hosts produced by other workers.

## Methods

**Figure 1:**
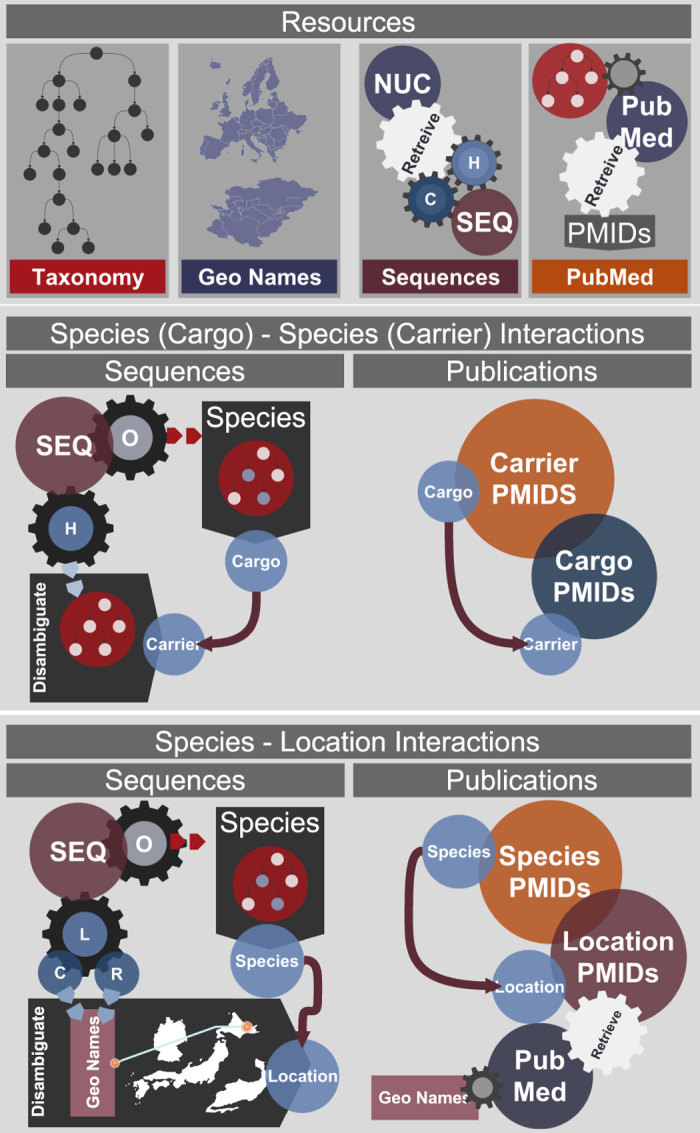
Overview of the methods of identifying species-species and species-location interactions. The first panel lists the resources used in a colour coded fashion. H refers to host and C to country tags in the sequence metadata. PMID is the PubMed unique identifier used in retrieving papers. The second panel explains the method of interrogating the evidence bases to extract species (cargo)-species (carrier) interactions. Species of sequenced organism (i.e., cargo) is first identified using the taxonomy tree, then the host tag in the sequence metadata is disambiguated using the taxonomic tree to identify the carrier species. Lists of PMIDs obtained for cargo and carrier species are intersected to provide additional evidence for the interactions extracted from the sequence metadata and to identify new relationships between cargo and carrier species discovered from the sequence metadata. The third panel illustrates the method of extracting species-location interactions from the evidence-base. First sequenced organisms and location information are extracted from sequence metadata. The species of sequenced organisms is then identified using the taxonomic tree. The location data (L) is split into country (C) and region (R) strings. Both are then disambiguated using the data gathered from GeoNames to obtain the country and region where the species was found. Geonames is also used to interrogate PubMed for papers about each country and region in the database. These are then intersected with species publications, the shared set is used as evidence for the species being found in a given location.

Open access, freely available web-based sources, such as the NCBI Taxonomy database (http://www.ncbi.nlm.nih.gov/taxonomy), NCBI Nucleotide database (http://www.ncbi.nlm.nih.gov/nuccore), PubMed citation and index (http://www.ncbi.nlm.nih.gov/pubmed) provide a source of valuable information that may not have been their primary focus. For instance, the descriptive metadata uploaded with genetic sequence submissions can be mined for the purposes of identifying interactions among species, including host-pathogen interactions, or the geographical distribution of the sequenced organism. Below we describe the processes by which raw data were obtained from these sources, and the methods by which interactions between organisms, or organisms and their geographical locations were extracted from these data. Figure 1 illustrates the overall process.

### Data repositories

#### Organisms, rankings and taxonomic hierarchy

856,031 organism scientific names, their unique identifiers (TaxID), taxonomic ranks and classifications were obtained from the NCBI Taxonomy database (http://www.ncbi.nlm.nih.gov/Taxonomy/). This dataset was manually supplemented with an additional 2,171 organisms of interest that were not found in the NCBI Taxonomy database.

The NCBI definition for ‘species’, ‘subspecies’ and ‘no rank’ (excluding viruses and viroids), was subsequently altered according to the following rules:

1. Where organism name contained numbers=>No rank.
2. Where organism name contained any of the following words (*unclassified*, *uncultured*, *var*)=>No rank.
3. Where word count=2=>Species.
4. Where word count ≥3=>Subspecies.

The application of the above rules resulted in the following changes: Species count decreased from 579,175 to 224,751. Subspecies count increased from 12,289 to 14,821. No rank count increased from 80,692 to 432,584. The remaining organisms were classified at or above genus level and were therefore excluded from the datasets described in this paper.

Taxonomic lineage relationships of the form Organism *A* is a parent of Organism *B* were also obtained from NCBI to replicate the hierarchical phylogenetic structure for the species, subspecies and no ranks listed above so that outputs can be obtained for species and higher taxonomic groups (for instance ‘flaviviruses’, ‘ruminants’). The 2,171 additional organisms were forced into the resulting tree, assigned to the most suitable parent nodes; where the correct parent was not found already in the tree, new nodes were added.

Some organisms were then linked to a collection of alternative names (e.g., common names, common misspelling, breeds and acronyms) that were collected from a variety of sources including textbooks. Additional care was given to humans and their domestic animals. Here, we focussed on 46 species of common domestic animals in Europe^9^. Where needed, the organisms were linked to sets of inclusion (AND) and exclusion (NOT) terms. These sets (alternative names, inclusion and exclusion terms) were utilised in disambiguating organism names and in retrieving publication metadata from PubMed as described in subsequent sections.

#### Geographical names

To enable the discovery of the geographical distribution of species a comprehensive dictionary of geographical names was built. First a list of countries and their alternative names was obtained from the GeoNames geographical database (http://www.geonames.org), and subsequently supplemented with the list of countries available in the Medical Subject Headings (MeSH) library (http://www.ncbi.nlm.nih.gov/mesh). For each country (particularly for larger countries), information about the country's administrative divisions was collected; State codes and acronyms for countries such as the United States, Brazil, and China were also added (e.g., NY for New York). For the purposes of the datasets described here only the first level administrative divisions (hereafter regions), were required; for these regions (e.g., home nations in UK, states in USA), extensive lists of major cities, natural features and unique place names were also obtained from GeoNames and other sources.

### Evidence curation

#### Nucleotide sequences

**Figure 2:**
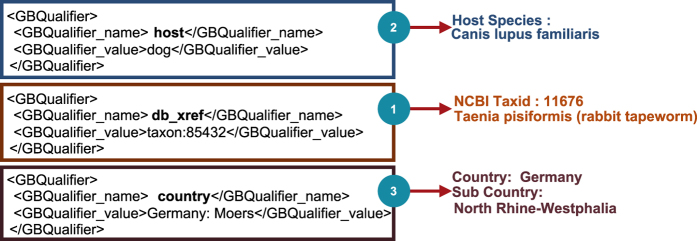
Example illustrating the information extracted from sequence metadata—sequence ID=158668169. .

The total of 39,238,061 nucleotide sequences' metadata files covering the period 1993–2012, were retrieved in XML format from NCBI Nucleotide Sequences database. The following data items were extracted (where available) from each metadata file (Fig. 2 illustrates this process):

1. NCBI TaxID: Using this identifier we were able to link 19,717,726 sequences with 171,967 corresponding species, 1,106,525 sequences with 10,989 subspecies, and 5,941,718 sequences with 245,532 no rank organisms.
2. Host: where available (7.1%) the host tag indicated the possibility of the sequenced organism being found *in* or *on* a host species.
3. Country: where available (17.5%) the country tag indicated the possibility of the organism being found in a certain geographical location that can be associated with a single country (and/or water body). 59.9% of sequences with country tag contained additional location information, such as the name or code of a state, a river, or a national park in which the organism was found.

#### Publications

Comprehensible search terms were automatically built using the three sets of names associated with each organism adhering to the following rule: ((Any of the organism names and alternative names) *And* (All of the inclusion terms)) *NOT* (any of the exclusion terms). Below is the search term generated for classical swine fever virus:

(‘classical swine fever virus’ [Text Word] OR ‘csfv’ [Text Word] OR ‘hog cholera virus’ [Text Word] OR ‘pestivirus type 2’ [Text Word] OR ‘swine fever virus’[Text Word]) NOT ‘african swine fever’ [Text Word]

6,473,167 citation metadata files were downloaded in XML format from the PubMed database. 6,028,487 of these files cited 7,463 species, 323,483 cited 208 subspecies and 674,836 cited 1,482 no rank organisms. Note that one paper may cite more than one organism.

### Identification of interactions

Using the data and evidence obtained and processed as discussed above, two types of interactions were identified: species-species interactions and species-geographic location interactions.

#### Species-species interactions

Species-species interactions indicate the possibility of one species (*Cargo G*) being found *in* or *on* another species (*Carrier A*). Many of these interactions are of the type: *Pathogen P was found in Host H*, however due to the nature of the underlying evidence we cannot assume all interactions to be of this type. Interactions can also be commensal (neither beneficial nor costly) or mutualistic (beneficial to both species), or vector-host. Additionally, an organism that is pathogenic to one host may be non-pathogenic in another so it is inappropriate to label the organism itself a pathogen; rather, it is interactions between species that are pathogenic or non-pathogenic. We therefore use a more generic terminology: *Cargoes are found in/on Carriers*. Cargoes are often pathogens and carriers are often hosts, but this is not always the case.

The identification of carrier-cargo interactions is a two steps process:

1. *Evidence extraction from nucleotide sequence metadata*: we have identified 2,706,620 metadata files (7.1% of the files obtained from NCBI) where information is provided for the *host* tag. Where the metadata for an organism includes an entry for the host tag, we infer a cargo-carrier interaction. These files were processed as follows:
  - Cargo species identification: sequenced organisms ranked above species-level were discarded from this dataset. Subspecies and no ranks were used to recursively identify their parent species in conjunction with the taxonomic tree. In other words, if the cargo is a subspecies, we store the interaction of the parent species with the carrier, not the subspecies itself.
  - Carrier species identification: the host tag was used to directly identify 73.6% of carriers to species level. For the remaining sequences a simple disambiguation algorithm was applied resulting in the identification of 94.5% of carriers. As with cargoes, sequenced organisms ranked above species level were discarded, and sequenced organisms ranked below species level (sub-species and no rank) were assigned to their parent species
2. *Evidence extraction from publications*: Having used the nucleotide database to define organisms as cargoes and carriers, we used this information to interrogate the publication metadata files obtained from PubMed. First, we retrieved all publication metadata files from PubMed for all cargoes and carriers identified above. Then, we intersected the two sets for common PubMed identifiers (i.e., finding papers which were in both sets). This enabled us to identify new combinations of carrier and cargo that were not apparent from the nucleotide evidence. Following a validation exercise^9^ a threshold of at least 5 papers was applied in including a publication-only interaction that was not backed up by sequence-based evidence.

**Figure 3:**
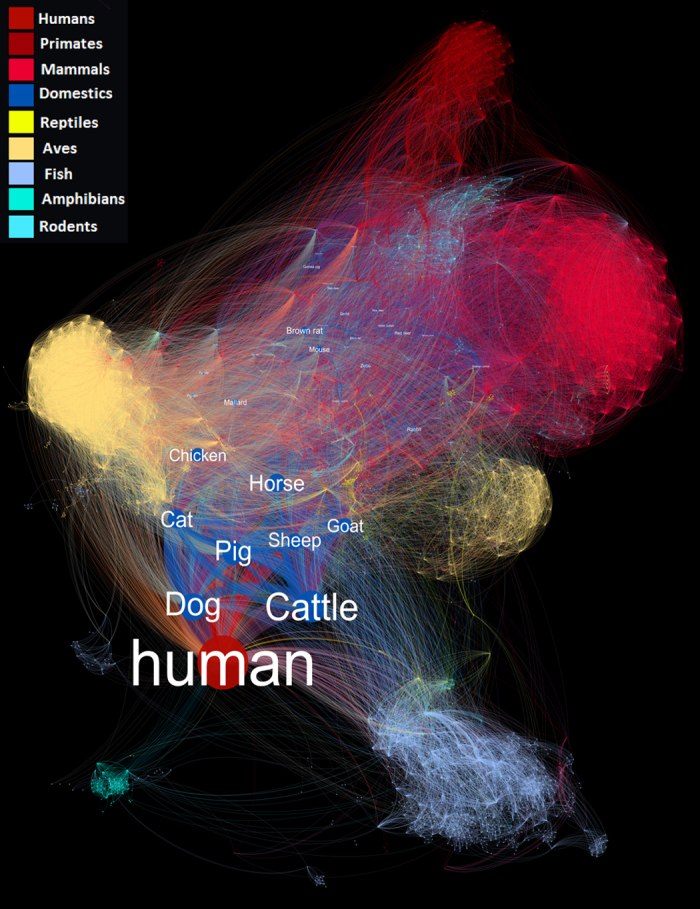
Shared pathogens between vertebrate species in Data Citation 1. Each node presents a vertebrate species. The size of the node is in proportion to the number of unique pathogen species found to interact with it. Edges between two nodes indicate they both share at least one possible pathogen species. The weight (thickness) of the edges is in proportion to the number of possible pathogen species shared between the two nodes. The location of each particular node corresponds to the size of all nodes in the graph and the weight of the edges linking this particular node with other nodes.

22,515 unique species interactions were thus generated between 6,314 carrier species and 8,905 cargo species. Figure 3 presents an example of how this dataset could be utilised in analysing and presenting potential pathogens (bacteria, viruses, fungi, helminth and protozoa species) shared between vertebrates species in Data Citation 1.

#### Species-location interactions

Location interactions indicate the possibility of a species being found in a certain location. Locations were interpreted at two levels: country C and region R. Regions correspond to first administrative divisions rather than geographical or natural regions (e.g., states (USA), departments (France), home nations (UK), etc.). Similarly to above, these interactions were extracted in two steps.

1. Evidence extraction from nucleotide sequences metadata: 6,714,520 metadata files where location information was provided (about 17.5% of the total), were processed as follows:
  - Species identification: sequenced organisms were processed in a similar way to cargoes in the previous subsection.
  - Location identification: The string within the country tag was assumed to adhere to the following format ‘Country: Location’, the typical format of items within the nucleotide database.
    - Country identification: the country part of the extracted strings was matched against our collection of geographical identifiers. Where the country was found to be a historical one (e.g., Yugoslavia) region information (if available) were used to identify the country (and where possible region), otherwise data were discarded. Water bodies (e.g., oceans and seas), were also discarded where no region substring was provided, or where the region substring was also a water body.
    - Region identification: countries without administrative divisions and small countries (e.g., Andorra) were excluded from this step. A region identification algorithm was applied. The algorithm splits the location string into substrings, each of which is matched against the collected location names from higher to lower ranked places (e.g., first administrative divisions, capitals, second administrative divisions, third administrative divisions, cities, towns, villages), and selecting the highest ranked match. Below are some examples:
      - *‘Italy: Milan’: C=Italy and R=Regione Lombardia.*
      - *‘USA: MA’: C=United States and R=Massachusetts.*
      - *‘China: Shantou’: C=China and R=Quangdong sheng.*
      - *‘United Kingdom: Yorkshire, Old Peak’: C=United Kingdom and R=England.*
2. Evidence extraction from publications: Suitable PubMed search terms were generated for countries and their regions, taking into account whether the country is in the MeSH library, and including the main geographical locations (such as region capitals, main cities, counties etc.) in the search terms, as per the following steps:
  - The country C is in the MeSH library:
    - A MeSH-based search term was generated to retrieve PubMed paper identifiers (PMIDs) for publications about C. We refer to this set of PMIDs as PMID_C_.
    - For each region R of C, a title and abstract only search term was generated using the region name, major cities and landmarks within the region. For instance, the following search term was used to retrieve publications about Scotland (*‘Scotland’ [title or abstract] or ‘Glasgow’ [title or abstract] or ‘Edinburgh’ [title or abstract]) or …..*.). We refer to this set of PMIDs as PMID_R_.
    - Only the PMIDs appearing in both sets were included when extracting information about regions within a country. We refer to this set as PMID_RC._
    - The set of PMIDs retrieved for each species was then intersected with PMID_C_ and PMID_RC_.
    - Where the results of the intersection contained five or more publications the interaction of species-country or species-region (in a country) was added to the database.
  - The country C is not in the MeSH library: an altered search term was used to look for the country in the title or abstract of the papers using the country's official name and the set of alternative names. Steps (a).ii-(a).v were then executed as described above.

In this way, 157,204 locations for 72,533 species were identified.

### The enhanced infectious disease database (EID2) database

The raw data curated in the above steps, and the identified interactions are stored in a web-fronted relational-database, the Enhanced Infectious Disease Database (EID2) (www. zoonosis. ac. uk/ EID2/). The database is continuously updated with new organisms, evidence and interactions. EID2 uses a 4-tier modular architecture separating the web front from the business logic and the database services following the S#arp Architecture model. EID2 is a web-based system, its user-interface UI is accessible via multiple web-browsers, and is supported by ASP.NET MVC framework from Microsoft Corporation. EID2 utilises and integrates various technologies such as Fluent Nhibernate for conversion-based, strongly typed mapping, and a number of technologies for visualisation and data-display. EID2 is freely accessible via a free of charge and simple registration procedure and subsequent login. In addition to the datasets presented in this paper, EID2 UI enables the user to access each of the evidence pieces on which the interactions were based; generate maps of the distribution of all organisms at both country level and region level, as well as access climate and other useful data.

## Data Records

### Species-species interactions

A summary of the species-species interactions is shown in Table 1. This table groups organisms (cargoes and carriers) in categories used by NCBI; we added an additional carrier category, domestic animals, which represents 46 species of domesticated mammals and birds in Europe^9^. Table 1 illustrates very clearly that while the vast majority of extracted interactions include the taxonomic groups most associated with pathogens (i.e., viruses, bacteria, fungi, protozoa and helminths), the automated procedures generate a number of other interactions, such as 297 interactions between cnidaria and fish species, and 2 between bryozoa and arthropod species.

The full species-species interactions dataset was uploaded, in csv format, to FigShare Data Citation 1. This dataset comprises the following fields:

1. Cargo: name of cargo species.
2. Cargo classification: the taxonomic classification of the cargo (e.g., bacteria, virus, etc.).
3. Carrier: name of carrier species.
4. Carrier classification: the taxonomic classification of the carrier (e.g., human, domestic, primates, mammals, etc.).
5. Sequences count: Total number of nucleotide sequences supporting the interaction.
6. Publication count: Total number of PubMed publications supporting the interaction.
7. Sequences: semi colon separated list of the nucleotide sequence identifiers (GIs) that can be used to retrieve these sequences from the NCBI Nucleotide database. For readability purposes this list was restricted to a maximum of 100 identifiers.
8. Publications: semi colon separated list of the PubMed citation identifiers (PMIDs) that can be used to retrieve these publications from the NCBI PubMed database. For readability purposes this list was restricted to a maximum of 100 identifiers.

### Species-location interactions

The generated species-location interactions dataset was uploaded, in csv format, to FigShare (Data Citation 2). This dataset comprises the following fields:

1. Species: name of species.
2. Species classification: the taxonomic classification of the species.
3. Country: official name of the country
4. Region: where available the official name of the region (sub-country) part of the location is provided.
5. Sequences count: similar to previous dataset.
6. Publication count: similar to previous dataset.
7. Sequences: similar to previous dataset.
8. Publications: similar to previous dataset.

## Technical Validation

### Validation of nucleotide evidence

We focus on validating the quality of the information extracted from the nucleotide sequences metadata, as this evidence base provide support to 94.36% of species interactions and 94.95% of location interactions. McIntyre *et al*.^9^ provides an in-depth validation of PubMed based interactions, particularly for location interactions in Europe.

#### Host disambiguation

host (carrier) species and sub-species were extracted from 24,656 unique strings in 1,074,943 nucleotide sequence metadata files. In 82.21% of the cases (in 74.47% of the files) no disambiguation was needed as the host tag contained the Latin name of the host. The remaining 4,412 host strings were manually checked against the collection of alternative names in the EID2 database. Following these two steps, a total of 99.44% host strings were correctly matched to a host (carrier) species. (99.63% when taking the number of sequence in which each string occurred into the equation).

#### Location disambiguation

Here we differentiate between country-only disambiguation and country and region disambiguation. Country level information was extracted from 2,216,652 nucleotide sequence metadata files (49% of total). Here, the verification was straightforwardly achieved by comparing the ‘country’ part of the string extracted from the sequence with the country names in the EID2 database. 99.99% of strings were correctly matched at country level (the mismatched values were former countries such as USSR, where no additional information was provided from which the present day country could be identified).

Country and region disambiguation were performed on 80,441 unique location strings extracted from 2,302,691 nucleotide sequence metadata files. In order to estimate the accuracy of our methods, we examined a sample of 13,150 unique country tags. The sample was randomly selected and the results manually checked. We found that 99.99% of the countries were correctly matched. Mismatched countries included ambiguous country tags such as *USA: Japan* and where the region and country belonged to two different countries. At regional level, we have found that 99.65% of the regions were correctly identified. Sample sizes for these validation exercises were estimated for accepted error of 1%, confidence level of 99%, and a conservative estimate of 50% failure.

### Validation of species-species interactions

#### Human pathogens

The species interaction data Data Citation 1 with *Homo sapiens* as the carrier (host) was compared against the 1,415 pathogens of humans listed in Taylor *et al*.^5^ 64.95% of human pathogens in Taylor *et al*.^5^ were included in our list, whereas 57.05% of human cargoes in our list were included in Taylor *et al*.^5^ Combining both sources resulted in 2,107 possible cargoes of humans. Table 2 lists the percentages shared per type of cargo.

#### Domestic mammals pathogens

A list of the pathogens infecting the domesticated mammal species as investigated in Cleaveland *et al.* ^3^ was obtained. Where the pathogen-host interactions were implicit, only the exclusive terms were selected (e.g., pathogen P1 infects all mammals, but not pathogen P2 infects some vertebrates). As the number of hosts in Cleaveland *et al*.^3^ is limited, we have focused on comparing the pathogens rather than the interactions. In other words, we have assumed that Data Citation 1 and Cleaveland *et al*.^3^ share a pathogen P if both datasets included an interaction between P and any of the hosts listed in Cleaveland *et al*.^3^ The percentages of shared cargoes were then calculated as follows: 67.10% of pathogens listed in Cleaveland *et al*.^3^ were found to be associated with the same species in Data Citation 1, and 59.15% of the cargoes extracted from Data Citation 1 for the mammals identified in Cleaveland *et al.* ^3^ were also found in Cleaveland *et al*.^3^ Combining both sources resulted in 1,339 possible cargoes for the domesticated mammals listed in Cleaveland *et al*.^3^ Table 3 lists the percentages shared per type of cargo.

#### Wild mammals parasites

A list of 5,142 interactions at species or sub-species level between wild mammals and their parasites was obtained from the Global Mammal Parasite Database (GMPD) (http://www.mammalparasites.org/)^10^. The list was then processed to identify unique interactions between wild mammals and their parasites, and only those with non-negative prevalence were taken into consideration. This is because the EID2 database is a presence only database, as the nature of the underlying evidence-base makes it hard to identify negative interactions of the type pathogen P is not prevalent in host H without further development of the algorithms used in extracting interactions from this evidence base. Following this step, 304 positive prevalence interactions between wild mammals and their parasites were identified from the GMPD database, and provided a basis for comparison with the species-interaction dataset discussed here.

The percentages of shared interactions between the species of wild mammals listed in GMPD and their cargoes were then calculated as follows: 64.02% of the unique interactions listed in GMPD were found in Data Citation 1, and 30.46% of the interactions found in Data Citation 1 for the mammals identified in GMPD were also shared with GMPD. Combining both sources resulted in 1984 unique interactions among 933 cargoes and 382 wild mammal hosts. Table 4 lists the percentages shared per type of cargo.

## Additional Information

**How to cite this article:** Wardeh, M. *et al.* Database of host-pathogen and related species interactions, and their global distribution. *Sci. Data* 2:150049 doi: 10.1038/sdata.2015.49 (2015).

## Supplementary Material



## Figures and Tables

**Table 1:** Summary of the species-species dataset (Data Citation 1)

	**algae**	**arthropod**	**bacteria**	**bryozoa**	**cnidaria**	**fungi**	**helminth**	**plants**	**mollusca**	**others**	**protozoa**	**segmented worm**	**viroid**	**virus**	**Total**
algae	0	0	36	0	0	0	0	0	0	0	0	0	0	2	38
amphibian	0	1	16	0	3	24	111	0	0	4	9	0	0	23	191
arthropod	5	294	1110	2	0	645	77	0	1	11	196	12	0	335	2688
aves	0	524	136	0	0	6	125	0	0	0	135	0	0	400	1326
bacteria	0	0	11	0	0	0	0	0	0	8	0	0	0	84	103
bryozoa	0	0	0	0	5	32	0	0	0	0	0	0	0	0	37
cnidaria	0	15	46	0	0	27	0	0	0	1	16	0	0	0	105
domestic	1	102	1215	0	0	227	556	0	0	12	458	0	0	563	3134
fish	0	47	588	0	297	82	1302	1	0	13	66	3	0	148	2547
fungi	0	0	73	0	0	1	1	0	0	0	1	0	0	23	99
helminth	0	0	91	0	0	12	0	0	2	0	0	0	0	7	112
human	1	19	878	0	0	311	148	0	0	0	70	0	0	204	1631
other mammals	0	110	298	0	1	114	412	0	1	7	202	0	0	406	1551
mollusca	1	29	138	0	0	2	109	0	0	8	39	0	0	7	333
plants	0	1506	2977	0	0	694	293	0	0	2	19	0	93	1597	7181
others	0	1	23	0	0	0	1	0	0	2	4	0	0	13	44
porifera	0	2	50	0	5	18	0	0	0	1	0	0	0	0	76
primate	0	11	26	0	0	19	36	0	0	0	104	0	0	231	427
protozoa	5	0	17	0	0	3	0	0	0	0	6	0	0	3	34
reptile	0	13	54	0	3	11	72	0	0	0	64	4	0	14	235
rodent	0	88	157	0	0	10	127	0	0	0	66	0	0	130	578
segmented worm	0	0	23	0	12	0	5	0	0	2	3	0	0	0	45
Total	13	2762	7963	2	326	2238	3375	1	4	71	1458	19	93	4190	22515
Columns are categories of cargoes, rows are categories of carriers.															

**Table 2:** Comparison between Taylor *et al*.^5^ and Data Citation 1 for human cargoes

**Cargoes**	**V**	**B**	**F**	**H**	**P**	**Totals**
in [5]	217	539	312	287	60	1415
in Data Citation 1	204	878	311	148	70	1611
in [5] and Data Citation 1	147	414	176	134	48	919
% [5] share with Data Citation 1	67.74	76.81	56.41	46.69	80.00	64.95
% Data Citation 1 share with [5]	72.06	47.15	56.59	1.67	68.57	57.05
Total Unique	274	1003	447	301	82	2107
V=Viruses, B=Bacteria, F=Fungi, H=Helminths, P=Protozoa^5^. classification of seven human pathogens has been changed to match that of NCBI, as recent evidence supports the latter sources' classification. These are (classification in brackets is taken from^5^): (1) Actinomadura pelletieri (B)-F, (2) Encephalitozoon cuniculi(F)-P, (3) Encephalitozoon hellem (F)-P, (4) Encephalitozoon intestinalis(F)-P, (5) Encephalitozoon bieneusi (F)-P, (6) Trachipleistophora hominis (F)-P, and (7) Vittaforma corneae (F)-P.						

**Table 3:** Comparison between Cleaveland *et al*.^3^ and Data Citation 1 for cargoes of domestic mammals

**Cargoes**	**V**	**B**	**F**	**H**	**P**	**Totals**
in [3]	147	228	88	349	193	915
in Data Citation 1	179	385	78	245	141	1038
in [3] and Data Citation 1	118	188	38	197	73	614
% [3] share with Data Citation 1	80.27	82.46	43.18	56.45	70.87	59.15
% Data Citation 1 share with [3]	65.92	47.59	48.72	80.41	51.77	59.15
Total Unique	208	435	128	397	171	1339
V=Viruses, B=Bacteria, F=Fungi, H=Helminths, P=Protozoa.						

**Table 4:** Comparison between GMPD and Data Citation 1 for wild mammals-cargoes interactions

**Interactions**	**V**	**B**	**F**	**H**	**P**	**A**	**Totals**
in GMPD	177	61	5	332	104	127	806
in Data Citation 1	486	395	51	283	284	95	1694
in GMPD and Data Citation 1	127	37	5	218	78	51	516
% GMPD share with Data Citation 1	71.75	60.66	100.00	65.66	75.00	40.16	64.02
% Data Citation 1 share with GMPD	26.13	9.37	9.80	56.92	27.46	53.68	30.46
Total Unique	536	419	51	497	310	171	1984
V=Viruses, B=Bacteria, F=Fungi, H=Helminths, P=Protozoa, A=Arthropod.							
